# System dynamics modelling of urbanization under energy constraints in China

**DOI:** 10.1038/s41598-020-66125-3

**Published:** 2020-06-19

**Authors:** Chaolin Gu, Xinyue Ye, Qiwen Cao, Weihua Guan, Chong Peng, Yutong Wu, Wei Zhai

**Affiliations:** 1 School of Architecture, Tsinghua University, Beijing, 100084 China; 2 Department of Informatics, New Jersey Institute of Technology, Newark, NJ 07102 USA; 3 School of Architecture, Tsinghua University, Beijing, 100084 China; 4 School of Geographical Sciences, Nanjing Normal University and Jiangsu Center for Collaborative Innovation in Geographical Information Resource Development and Application, Nanjing, 210023 China; 5 School of Architecture and Urban Planning, Huazhong University of Science and Technology and Hubei Urbanization Engineering Technology Research Center, Wuhan, 430074 China; 6 School of Landscape Architecture and Planning, University of Florida, Gainesville, FL 32611 USA

**Keywords:** Energy and society, Socioeconomic scenarios, Sustainability

## Abstract

The rapid urbanization in China has been associated with a growing hunger for energy consumption and steadily-increasing CO_2_ emissions. In this paper, an integrated system dynamics model composed of four sub-models is developed to simulate the urbanization and energy consumption in China from 1998 to 2050. Three scenarios are provided: accelerated economic development, emission reduction constraint, and low-carbon oriented. The result reveals that rapid economic growth and sufficient energy supply will foster China’s urbanization in all three scenarios. Under the low carbon transition scenario, China’s urbanization rate is expected to reach 76.41% in 2050, both reducing carbon emissions and promoting eco-friendly development. All three scenarios witness a dramatic growth of residential energy consumption and a steady increase of industrial energy consumption. China still has a long way to achieve the low-carbon transition goal. China should promote renewable resources and energy, pursue a low-carbon lifestyle, and reduce energy intensity over the next few decades.

## Introduction

There is a long and rich history of research exploring the association between energy demand and urbanization^1–3^. Energy consumption, and its socioeconomic and environmental impacts, have imposed a critical influence on urban sustainability^1^. At the same time, the effects of urbanization on energy consumption vary across scales and over countries^4–6^. China's dramatic urbanization and the associated energy demand as well as the pressure of CO2 emitted by such energy consumption is a major global scientific issue since this century^7–9^. Previous research in China has often focused on a specific field or aspect, such as urbanization theory^10^, urbanization procedure^11^, energy policy^12^, energy growth^13^, CO_2_ emission^14^, climate change^15^, and public health^16^.

Although urban expansion would accelerate energy consumption^17–19^, the automotive fuel use tends to correlate negatively with the urbanization level due to economies of scale^20–22^. For instance, more urbanized areas in Canada consumed lower per capita energy^23^. Additionally, total energy consumption of rural households is larger than that of urban households, because more than 85% of rural households use inefficient solid fuel^24^. According to Customer Data Platform, 275 cities worldwide use hydropower, 189 cities use wind power, and 184 cities use solar photovoltaic power. At least 100 cities use renewable energy to achieve 70% of electricity, such as Seattle, Oslo, Vancouver, and Nairobi. In the United States, 58 cities, including Atlanta and San Diego, have plans to transition to 100% clean and renewable energy. With the existing technology and energy storage, it is expected to achieve 100% renewable energy supply worldwide and achieve zero carbon emissions by 2025. The global power generation structure in 2050 is expected as below: solar photovoltaic (69%), wind power (18%), hydropower (8%), and biomass (2%), with energy storage batteries covering 31% of power demand. It is noted that most of the world's largest dams and power plants are located in China, while Yunnan Province and Guizhou Province contribute 30% of China's hydropower. The Jinsha River alone has a 9-level dam. The electricity generated by these hydropower is directly transmitted to Guangdong and Hong Kong through the high-voltage grid. Due to the abundant hydropower resources available for cooling, Guizhou Province has recently become China’s most important data center base. In 2018, Apple Corporation built the iCloud data center in Guizhou. Almost every large IT company of China has set up data centers in Guizhou, including Alibaba, Huawei, and China Mobile.

Urbanization is a non-linear open complex system, with multiple subsystems dynamically interacting with each other^25–27^. Dramatic urbanization under energy constraints is challenging for China, which has topped all the countries in population, energy consumption (since 2010), and CO_2_ emissions (since 2008)^27^. To systematically examine the causality mechanism between energy consumption and urbanization, the SD (system dynamics) model has been widely used in the domains such as energy consumption^28^, energy policy^29,30^, energy efficiency^31^, carbon emission^32,33^, and energy industry^34,35^. The SD model, Monte Carlo simulation, and Hornberger-Spear-Young (HSY) algorithm were employed to analyze the urbanization patterns with the limit of energy and environmental resources^36^. In addition, the baseline scenario was simulated from 2005 to 2020 using the SD model of urbanization and the energy consumption complex system^28^. The SD model was also developed using the STELLA platform to model the energy consumption and CO_2_ emission trends^33^. To dynamically predict future urban development trends under various scenarios has very important scientific values for the policy formulation.

## Methodology

Urbanization facilitates socioeconomic and industrial transitions^37^. However, urbanization also has negative impacts on social equity, public health, and the environment^38–41^. Though the effects of energy demand and supply on the environment have been extensively investigated^42–47^, the causality relationship between urbanization and energy demand/supply/environmental impacts has not been conducted from the SD perspective. Admittedly, the effects of urbanization on energy consumption have been examined by computable general equilibrium (CGE) and regression models^48–51^. However, the causality relationship between urbanization and energy consumption is difficult to be reflected using these methods^40^. Therefore, this paper makes important contributions to the literature by constructing the SD model integrating the above three elements from the causal loop perspective, through setting various energy constraint scenarios for policy implications in China. A SD model is conducted as below: (1) define the problem; (2) establish a system’s functional model framework; (3) identify the causal relationship of the model and a system flowchart of the feedback loop; (4) design the equations and parameters of each variable; (5) test the validity of the model; (6) modify the model parameters for the improved performance; (7) evaluate various policy implications based on the simulation results^52–55^.

## The urbanization-energy SD model

### Description of the model

Since the Industrial Revolution, cities have shifted from center of politics and trade to the consumption and production. Population-industry-capital-technology-urbanization constitutes a city’s social-economic system. Entering the period of manufacturing-led urban development, energy and urban development are closely related, due to the heavy use of fossil energy resulting in SO_2_ and CO_2_ emissions.

**Figure 1:**
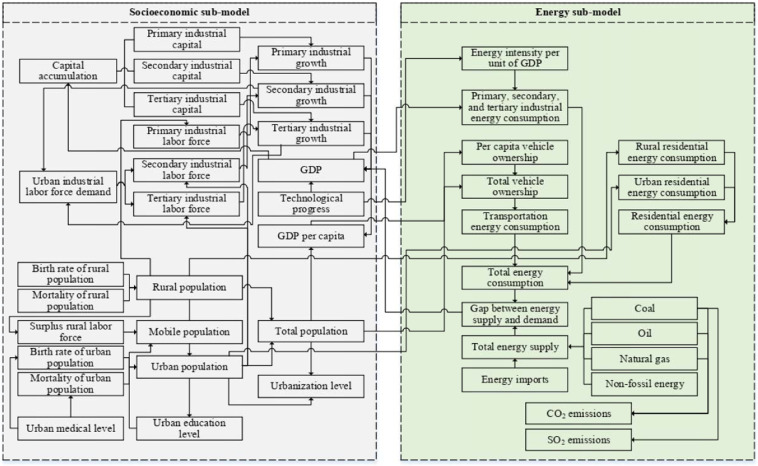
The causal loop diagram of urban socio-economic and energy system (drawn using Microsoft Visio Professional 2013: https://microsoft-visio.en.softonic.com/).

The system (Fig. 1) is comprised of two components: the socioeconomic subsystem and the energy supply-demand-environmental subsystem. The socioeconomic subsystem consists of capital, population, urbanization, industries, and public services. The energy supply-demand-environmental subsystem includes three parts: (1) total energy sources: coal, oil, natural gas, and non-fossil energy; (2) energy consumptions: industrial energy consumption, residential energy consumption, and energy for transportation; (3) energy-environment indexes: energy intensity per unit of GDP, CO_2_ and SO_2_ emissions from energy consumption. In an SD model, stocks can be calculated with the integration of their flows, described by Eq. (1). After defining stocks, it is then possible to decide the flows and auxiliaries^56^. The stock and flow diagram is the algebraic representation of the model based on the identified causal loops.

1 $$Stock(t)={\int }_{t0}^{t}[Inflows(s)-Outflows(s)]ds+Stock(t0)$$

#### Socioeconomic sub-model

Economic growth and population migration are closely associated with energy consumption^57^. China’s urbanization has been facilitated by rural labor force surplus, urban industrial development, as well as the gap between high level of industrialization/urbanization as well as high quality of life and energy shortage as well as low quality of ecological environment^55^. Based on the Cobb-Douglas Production Function, the socioeconomic sub-model examines the interactions among economic growth, labor productivity, and the labor force demands. There are nine indicators selected as the stock variables, including the output value of the primary industry, output value of the secondary industry, output value of the tertiary industry, total capital stock, labor force of the secondary industry, labor force of the tertiary industry, input of the agricultural labor force, rural population, and urban population.

The relationship between labor force input and product output can be expressed as^58^:

2 $$G=L\cdot P$$

where G denotes gross national product (or gross national income), L represents labor force input, and P represents labor productivity. By transposition, we get Eq. (3).

3 $$L=G/P$$

Equation (3) indicates that the labor force input, or labor force demand (job opportunity), is a function of the gross national product (gross national income) and labor productivity. Through derivation, we get Eq. (4):

4 $$e=g-p$$

where the growth rate of labor force demand *e* is equal to the difference between the growth rate of gross national product *g* and the growth rate of labor productivity *p*. Since the growth rate of labor force demand is equal to the quotient of added labor force demand divided by the labor force demand of the previous perio the labor force of the primary and tertiaryd, the added labor force demand *dL* can be expressed by Eq. (5):

5 $$L=(g-p){L}_{0}$$

As the total labor force demand for a current period is the sum of *dL* and *L*_0_, the total labor force demand L in Eq. (3) *L *= *G/P* should be expressed as:

6 $$L=(1+g-p){L}_{0}$$

By following this procedure, we can have the labor force demand of the secondary industry ${L}_{2}^{t}$ and the tertiary industry ${L}_{3}^{t}$ expressed by Eqs. (7, 8):

7 $${L}_{2}^{t}-{L}_{2}\times (1+{g}_{2}-{p}_{2})$$

8 $${L}_{3}^{t}-{L}_{3}\times (1+{g}_{3}-{p}_{3})$$

where *L*_2_ and *L*_3_ are the labor force of the primary and tertiary industry respectively; *g*_2_ and *g*_3_ are the growth rate of output value of both the secondary and tertiary industry respectively; *p*_2_ and *p*_3_ are the growth rate of the labor force of the secondary and tertiary industry respectively.

With the advance of agricultural labor productivity, agricultural population will be partially transferred to non-agricultural industries. The net rate of agricultural labor force transfer is the difference between the growth rate of agricultural labor force supply and demand. So the net rate of agricultural labor force transfer is decided by agricultural scale and agricultural labor productivity. Thus we have Eqs. (9) and (10):

9 $${L}_{1}^{t}={G}_{1}/({P}_{1}\times (1+{p}_{1}))$$

where ${L}_{1}^{t}$ is agricultural labor force demand; *G*_1_ is agricultural output value, P_1_ is agricultural labor productivity, and *p*_1_ is the growth rate of agricultural labor productivity.

10 $$L{q}_{1}=L{q}_{1}-({L}_{1}^{t}/({L}_{1}-d{L}_{1})-1)$$

where ${L}_{q1}$ is the rate of agricultural labor migration; ${L}_{g1}$ is the growth rate of the agricultural labor force; *L*_1_ is agricultural labor force input; and *dL*_1_ is the annual added agricultural labor force.

The relationship between fixed capital stock (denoted by K) and total output (denoted by Y) can be expressed using Eq. (11):

11 $$dY/dK=Y/K$$

Then, by transposition, we get

12 $$K=Y(dK/dY)$$

where *Y* is GDP; *dY* is the annual change of GDP; *K* is fixed capital stock; *dK* is the annual net increment of fix capital stock.

**Figure 2:**
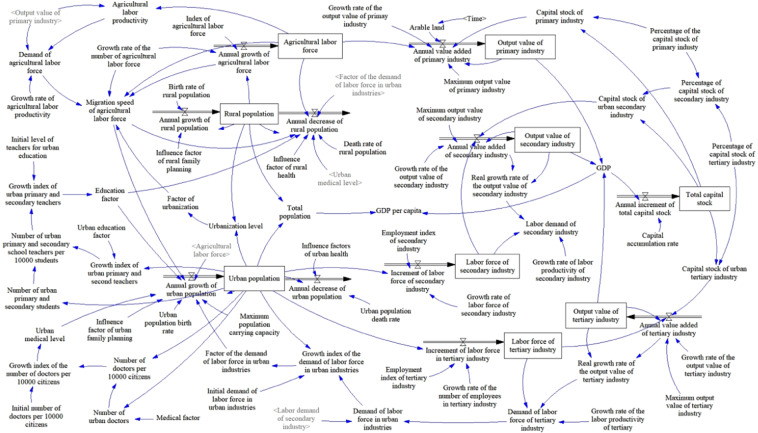
Socioeconomic sub-model of Chinese urbanization-energy SD model (drawn using Vensim PLE 7.2: http://vensim.com/).

These elements can be briefly described in Fig. 2 regarding their relationship. Appendix A lists these variables and equations in the socioeconomic sub-model.

#### Energy supply-demand-environmental sub-model

Total primary energy supply is represented by indigenous energy production and balance between imports and exports, including coal, petroleum, natural gas, and non-fossil energy^59^. The energy supply can be defined as:

13 $$TES=\mathop{\sum }\limits_{j=1}^{4}E{P}_{j}\times (1+EP{R}_{j})+EIE$$

**Figure 3:**
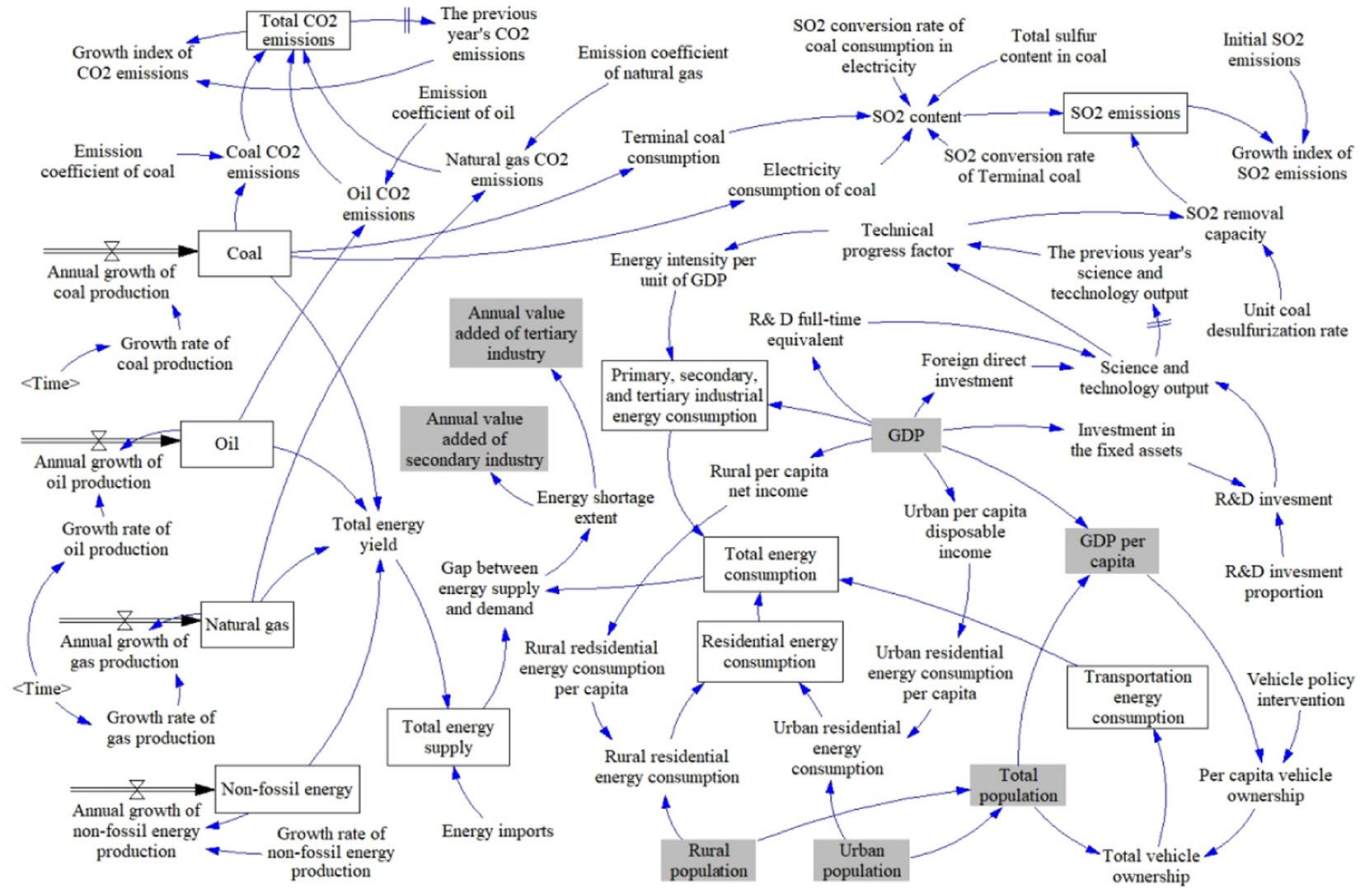
Energy supply-demand-environmental sub-model of Chinese urbanization-energy SD model (drawn using Vensim PLE 7.2: http://vensim.com/).

Where TES is the total energy supply; *EP*_*j*_ is the jth type of energy production; *EPR*_*j*_ is the growth rate of the jth type of energy production; *EIE* is the energy import and export balance. Energy production includes the production of coal, oil and natural gas, and non-fossil energy. Non-fossil energy includes new energy and renewable energy, such as wind, solar, hydro, biomass, geothermal, ocean energy and nuclear energy. Since fossil energy and non-fossil energy cannot be converted into unified basic energy, the fossil fuel and non-fossil energy are calculated separately (Fig. 3).

Total energy demand is composed of the energy consumption from the industry, transportation, and residential sectors^60^. Transportation, warehousing, and postal services are considered as mobile sources, which are calculated in the transportation sector^61^, differing from those of the terminal energy consumption in the industry. Energy consumption of the production can be derived as the amount of the energy consumption from the primary, secondary, and tertiary industries as well as their energy intensity (Fig. 3). Energy intensity can be calculated as energy consumed per unit of GDP, reflecting that the industrial energy consumption rely on economic development and technology innovation^27,62,63^. Based on the 1998–2015 data, energy intensity is measured (Appendix A). Energy consumption from the industry sector is calculated as below:

14 $$IEC=\mathop{\sum }\limits_{i=1}^{3}AV\times EI$$

15 $$EI=f(f(RDI,FTE,FDI),GPC)$$

where the IEC is the industrial energy consumption; EI is the energy consumption per unit of industrial value added; RDI is the R&D investment; FTE is the R&D full-time equivalent; FDI is foreign direct investment; GPC is the GDP per capita.

Transportation includes railway, road, waterway, air, and pipeline, as well as loading and unloading services. Since these data are unavailable, the transportation energy consumption is calculated using the consumption of vehicles. Macroeconomic conditions and demographic factors affect the vehicle demand. China’s vehicle stock is estimated by the Gompertz model, explaining the growth of the vehicle ownership (vehicle/population) as a function of per capita income^40^. Energy demand in the transport sector is modeled as a product of quantity of vehicles, activity level, and fuel efficiency as below:

16 $$VEC=P\times (\phi \times {e}^{\delta \times {e}^{(\partial \cdot A)}})\times AVT\times CFE$$

where VEC is the transportation energy consumption; P is the population; AVT is the average annual driving distance of vehicles; CFE is the fuel consumption per unit distance.

Increasing incomes of residents have been accelerating the popularity of durable consumables, such as electrical household appliances and cars as well as housing space, leading to the soaring energy consumption of domestic sectors^41^. Based on the correlation between per capita residential energy consumption and income (Appendix A), the residential energy demand can be defined as:

17 $$REC=URP\times f(UCI)+RUP\times f(RCI)$$

Where REC is the residential energy consumption; URP and RUP are the urban and rural population, respectively; UCI is the urban disposable income per capita; RCI is the rural net income per capita; others are parameters to be estimated.

Energy consumption produces a number of greenhouse gases and pollutants, especially CO_2_ and SO_2_, which have negatively influenced environmental quality^64^. Coal consumption has contributed to most of SO_2_ emissions in China^65^ (Fig. 3). The material balance method^52^ is adopted to calculate the SO_2_ emissions through the coal consumption as below:

18 $$PFS=2\times (ECC\times SC{O}_{1}+TCC\times SC{O}_{2})\times TSC\times (1-NSO)$$

where *PFS* is the SO_2_ emissions; *ECC* is electricity coal consumption; *TCC* is the terminal coal consumption; *SCO*_1_ and *SCO*_2_ are the SO_2_ conversion rate of coal consumption in electricity and terminal respectively; *TSF* is the total sulfur content in coal; *NSO* is the desulfurization efficiency.

The CO_2_ emissions are estimated based on energy consumption and carbon emission coefficient. The carbon emission coefficient of various energy types adopts the corrected value using the 2006 IPCC recommended method^12,66^. CO_2_ emissions are projected as below:

19 $$TCE=\mathop{\sum }\limits_{j=1}^{3}(E{P}_{j}\times CE{F}_{j})$$

where *TCE* is the energy-related CO_2_ emission in terms of ton of standard coal equivalent; *EP*_*j*_ is the jth type of energy consumption; *CEF*_*j*_ is the CO_2_ emission coefficient of the *j*th type of energy.

141 key performance indicators of the sub-systems are depicted in Appendix A. The model contains three types of variables and one type of parameter, including stock variables, rate variables, auxiliary variables, and constants.

### Data

The data is mainly from China Compendium of Statistics 1949–2008, China Statistical Yearbook, China Energy Statistical Yearbook, National Environment Statistic Communique, and the survey data of government agencies, including the National Development and Reform Commission of China, Ministry of Transport of China, and the National Energy Administration of China. The average travel distance of motor vehicles and fuel data are from China Car Energy Outlook 2012. Since the data of Hong Kong, Macau, and Taiwan are unavailable, the SD model only covers Mainland China. The subsystems are illustrated in the following sections, and the variables and equations in this model are detailed in Appendix A. The study period is from 1998 to 2050. The software Vensim is used for model building.

### Parameter setting

In addition to the variables, the constants are included in the SD model through the initial values of variables and parameters. Most parameters are calculated by the methods of historical data averaging, extrapolation of development trends, table functions (in Appendix A), and the Cobb-Douglas Production Function. In addition, because many factors affect China’s urbanization, and the interaction mechanism is very complicated, this article uses grey relational analysis^67^. GM (1,1) is a long-term forecasting Grey Model (GM), which mainly solves the generation sequence with exponential change law. The calculation steps of GM (1, 1) are as follows:

First, we select the system reference sequence and comparison sequence ${X}_{i}=\{{X}_{i}(t),t=1,2,\cdots n\}$ (i = 1, 2, …, n); the reference sequence and comparison sequence are initialized to make them dimensionless and normalized. Second, we calculate the gray correlation coefficient between the reference sequence and comparison sequence at time t = j:

20 $${\xi }_{1}=\frac{\frac{\min }{i}\frac{\min }{j}|{X}_{0}(j){X}_{i}(j)+\alpha \frac{\max }{i}\frac{\max }{j}{X}_{0}(j)-{X}_{i}(j)}{|{X}_{0}(j){X}_{i}(j)+\alpha \frac{\max }{i}\frac{\max }{j}{X}_{0}(j)-{X}_{i}(j)}$$

where *α* is the resolution factor between 0 and 1, usually set to be 0.5. Lastly, the correlation is calculated as:

21 $${r}_{i}=\frac{1}{n}\mathop{\sum }\limits_{j-1}^{n}{\xi }_{i}(j)$$

The energy data is in grams of the standard coal equivalent. To minimize the price effect from a data series, the constant prices in 1990 are adopted as the initial values of the output value and capital variables. The constants and their initial values are shown in Table 1.

**Table 1:** Parameters of the constants in the SD model.

Methods	Variables	Parameter	Unit
From the average of historical data in statistical yearbook	Birth rate of rural population (source: National Population Statistics of China)	1.4	%
	Capital accumulation rate (source: China Statistical Yearbook of Fixed Assets Investment, China Statistical Yearbook)	49	%
	Death rate of rural population (source: National Population Statistics of China)	0.6	%
	R&D investment proportion (source: China Statistical Yearbook)	2.64	%
	Growth rate of the output value of the primary industry (source: China Statistical Yearbook)	5.52	%
	Growth rate of the output value of the secondary industry (source: China Statistical Yearbook)	9.4	%
	Growth rate of the output value of the tertiary industry (source: China Statistical Yearbook)	11.7	%
	Urban population birth rate (source: National Population Statistics of China)	1.11	%
	Urban population death rate (source: National Population Statistics of China)	0.52	%
	Employment index of the secondary industry (source: China Statistical Yearbook, China Population and Employment Statistical Yearbook)	0.333	—
	Employment index of the tertiary industry (source: China Statistical Yearbook, China Population and Employment Statistical Yearbook)	0.4137	—
	Growth rate of the agricultural labor force (source: China Statistical Yearbook, China Population and Employment Statistical Yearbook)	−1.96	%
	Growth rate of the number of employees in the tertiary industry (source: China Statistical Yearbook, China Population and Employment Statistical Yearbook)	2.76	%
	Growth rate of the labor force of the secondary industry (source: China Statistical Yearbook, China Population and Employment Statistical Yearbook)	2.33	%
	Growth rate of the labor productivity of the secondary industry (source: China Statistical Yearbook, China Population and Employment Statistical Yearbook)	6.9	%
	Growth rate of the labor productivity of the tertiary industry (source: China Statistical Yearbook, China Population and Employment Statistical Yearbook)	8.7	%
	Growth rate of agricultural labor productivity (source: China Statistical Yearbook, China Population and Employment Statistical Yearbook)	7.7	%
	Percentage of capital stock of the tertiary industry (source: China Statistical Yearbook, China Population and Employment Statistical Yearbook)	55	%
	Percentage of capital stock of the primary industry (source: China Statistical Yearbook, China Population and Employment Statistical Yearbook)	1.8	%
	Determinants of rural family planning (source: China Statistical Yearbook, National Population Statistics of China)	1.15	—
	Determinants of urban family planning (source: China Statistical Yearbook, National Population Statistics of China)	1.05	—
	Determinants of rural health (source: China Statistical Yearbook, National Population Statistics of China)	0.95	—
	Determinants of urban health (source: China Statistical Yearbook, National Population Statistics of China)	0.92	—
	Medical determinants (source: China Statistical Yearbook, National Population Statistics of China)	0.98	—
From the government documents	SO_2_ conversion rate of coal consumption in thermal power	0.9	—
	SO_2_ conversion rate of terminal coal	0.8	—
	Total sulfur content in coal	1.2	%

### Validation and sensitivity test

Validation of the SD model is performed using two approaches. A structural verification of the model seeks to determine whether it reflects the real world accurately. Another sensitivity tests focuses on the model behavior during execution, and assesses the degree of confidence.

#### Structural validity

Structural validity of the SD model is tested in terms of dimensional consistency^68^. Structural validation is performed using structural verification and extreme values. Based on the real data, preliminary values of the model’s variables are examined and their rationality is ascertained. Then the above parameters are entered into the model as the stocks flow test. The error rate between real data and simulated data demonstrates the reliability of the model (Table 2).

**Table 2:** Stocks flow test of the Model (variable source: China Statistical Yearbook).

Variables	Error rate (%)	Variables	Error rate (%)
Urbanization level	0.813	Total population	0.510
GDP (price in 1990)	2.673	Output value of the primary industry	7.298
Output value of the secondary industry	5.530	Output value of the tertiary industry	2.061
Production of coal	9.673	Production of petroleum	1.575
Production of natural gas	7.331	Production of non-fossil energy	3.644
Industry energy consumption	7.038	Residential energy consumption	3.810
Transportation energy consumption	5.191	Total energy consumption	6.125

Note: Error rate between real data and simulated data (%).

Table 2 demonstrates that the error rate between real data and simulated data of the 14 key variables selected from the model are mainly below 10%. More explanations are in 4.2. In addition, Table 2 in Appendix B lists the details of the validation outcomes.

#### Sensitivity test

Sensitivity test refers to examine how changing the threshold of parameters might affect the output of the model. A robust model should be insensitive to the changes of most parameters. Therefore, after the Energy-Urbanization SD model has passed the structural validity test based on historical data, sensitivity tests are conducted^69^.

The sensitivity level of a parameter is measured as below:

22 $${S}_{Q}=|\frac{\Delta {Q}_{(t)}}{{Q}_{(t)}}\cdot \frac{{X}_{(t)}}{\Delta {X}_{(t)}}|$$

23 $$S=\frac{1}{n}\mathop{\sum }\limits_{i=1}^{n}{S}_{{Q}_{i}}$$

where t is the time; ${Q}_{(t)}$ and..are the values of $Q$ and $X$at time t, respectively; ${S}_{Q}$ is the sensitivity value of the level variable $Q$ to parameter $X$; .. and $\varDelta {X}_{(t)}$are the change values of $Q$ and $X$ at time t, respectively; $n$ is the number of level variables; ${S}_{{Q}_{i}}$ is the sensitivity value of ${Q}_{i}$ and $S$ is the average sensitivity level of parameter *X*.

14 key variables are selected from the model to test the sensitivity of changes in urbanization level to these 14 parameters^52,53^. From 1998 to 2050, each parameter increases or decreases by 10% year by year, and such impact on the level of urbanization is examined. According to Eq. (8), each variable can obtain two sensitivity values, so the average of 28 sensitivity values can represent the sensitivity of urbanization level to a specific parameter. Using Eq. (9) to calculate the average sensitivity of 14 variables to a specific parameter can lead to a total of 28 values. The results are shown in Table 3.

**Table 3:** The results of sensitivity analysis.

Variables	+10%	−10%	Variables	+10%	−10%
Birth rate of the rural population	11.56	11.05	Birth rate of the urban population	3.81	3.89
Determinants of rural family planning	11.56	11.05	Determinants of urban family planning	3.81	3.89
Education factor	23.80	6.05	Growth rate of the labor force of the primary industry	8.34	8.07
Growth rate of the labor force of the secondary industry	0.35	0.36	Growth rate of the labor force of the tertiary industry	0.63	0.67
Growth rate of coal production	1.42	1.38	Growth rate of petroleum production	0.11	0.11
Growth rate of natural gas production	0.34	0.33	Growth rate of non-fossil energy	0.41	0.49
The SO2 removal rate of per unit coal	0.45	0.41	Proportion of R&D investment	0.01	0.01

Three among the 14 variables show a sensitivity value larger than 10%, including the birth rate of the rural population, determinants of rural family planning, and education. Hence, the system is insensitive to changes of most parameters. In summary, through the structural validity analysis and sensitivity test, this model proves to be robust and can be used for scenario simulation of the real system.

## Scenario Simulation and Results

### Scenario setting

To compare the evolution of China’s urbanization, energy and environmental conditions under different scenarios from 2015 to 2050, eight parameters are used: primary industry growth, secondary industry growth, tertiary industry growth, coal production growth rate, oil production growth rate, natural gas production growth rate, non-fossil energy production growth rate, and the number of fuel-driven vehicles. Three development modes are designed: the accelerated economic development scenario (AED), the emission reduction constraint scenario (ERC), and the low-carbon oriented development scenario (LOD), as shown in Table 2. The AED scenario reflects China’s high energy consumption path; the ERC scenario shows a significant reduction in fossil energy but no other energy policies; the LOD scenario is the active adjustment of the energy structure with industrial restructuring through largely increasing non-fossil energy supply while significantly reducing fossil energy (Table 4). The initial value of each variable is set to that in the year of 2015, with one year being the temporal interval.

**Table 4:** Parameters of three scenarios.

Parameter		Scenario		
		AED	ERC	LOD
Growth rate of output	primary industry	4%	3%	3%
	secondary industry	7%	5%	5%
	tertiary industry	8%	6%	8%
Growth rate of the production of fossil energy	coal	Adopt historical data	0.47% from 2015–2030 −0.98% from 2031–2050	0.47% from 2015–2030 −0.98% from 2031–2050
	petroleum	Adopt historical data	4.75% from 2015–2030 3.61% from 2031–2050	4.75% from 2015–2030 3.61% from 2031–2050
	natural gas	Adopt historical data	5.67% from 2015–2030 0.65% from 2031–2050	5.67% from 2015–2030 0.65% from 2031–2050
Growth rate of the production of non-fossil energy		Adopt historical data	Constant	7.64%
Motor vehicle policy		Adopt historical data	Constant	phase out production and sales of fossil fuel cars by 2035

#### Accelerated economic development (AED) scenario

The emerging and developing economies have been witnessing extraordinary paces of urbanization associated with rocketing energy consumption^70^. Energy is essential for the economic growth and urbanization^71^. The production of coal, oil, natural gas, and non-fossil energy will further rise under the AED scenario. Fossil energy is still the main source of energy supply for China. The energy supply will increase tremendously with the growth of energy demand. Since 2015, China has entered a new normal economic development model to adjust economic structure. For this reason, it is expected that the growth rate of GDP will not exceed 8% in the near future^72,73^. The growth rate of the primary industry, the secondary industry, and the tertiary industry is set according to the existing research on China’s economic growth forecast, while the other five variables use China’s average historical growth rate from 1998 to 2015. Therefore, the growth rates of the primary, secondary, and tertiary industrial output-values are estimated to be 4%, 7%, and 8%, respectively.

#### Emission reduction constraint (ERC) scenario

It is urgent to reduce the dependence on fossil fuels through strict environmental and energy policies, especially considering the limited reserves of China’s fossil energy^74^. The Chinese Academy of Engineering’s Medium- and Long-Term Energy Development Strategy Research Project Group uses a comprehensive evaluation model to predict that energy consumption in 2050 will be 66.57 * 108tec in the baseline scenario, 52.50 * 108tec in the low-carbon scenario, and 50.14 * 108tec under the low-carbon scenario^75^. Many other studies predict that total energy consumption in 2050 will be between 51.89 * 108tec ~ 89.33 * 108tec^76–79^. Hence, the peak of fossil energy consumption and capacity in China will be about 32.76-49.35*10^8^tec between 2030 and 2035^80,81^. Taking into account the predictions of total energy consumption and the trend of the proportion of fossil energy in total energy, the peak of fossil energy production will be 38*10^8^tec in 2030, and then decline to 29.5*10^8^ tec in 2050 under the ERC scenario. The growth rates of coal, oil, and natural gas are set based on this assumption. As the supply of fossil fuels declines, the growth rate of the primary, secondary, and tertiary industrial output-values will also slow down, in order to control the energy demand and achieve the balance of energy supply and demand.

#### Low-carbon oriented development (LOD) scenario

The LOD scenario involves the optimization of energy structure to lower the reliance on the fossil energy^82^. The growth rate of fossil energy production has been greatly reduced, but a high growth rate of non-fossil energy has been set to ensure that the total energy supply can meet socio-economic development needs. According to the China 2050 High Renewable Energy Penetration Scenario and Roadmap Study by the Energy Research Institute of the National Development and Reform Commission, renewable energy will be 60% of energy consumption by 2050^83^.

Transportation is a main source for energy consumption and carbon emissions, so low-carbon travel is advocated. UK and France have announced a total ban on gasoline and diesel-powered cars from 2040. In Environmental Protection Project 2030, Germany requires strict control of automobile CO_2_ emissions and only sells zero-emission vehicles from 2030. In 2015, China has become the world’s largest new energy vehicle production and sales market. In the future, new energy vehicles will gradually replace fuel vehicles. The ban on the sale of fuel vehicles in China is expected to be implemented in 2035^84^. Therefore, this scenario will regulate the number of motor vehicles. Moreover, China might optimize the industrial structure by accelerating the service industry.

### Results 1

This SD model has been verified using the data of 1998-2015. Table 2 of Appendix B shows: (1) Urbanization level and Population: the gap between the actual value and simulation is less than 1.0%; (2) Output value of tertiary industry (constant prices in 1990): except that the error in 2013 is more than 6.5%, the others are within 3.5%. The foundation of tertiary industry was weak, and the development of the Producer Services industry was relatively slow. They continued to develop with the promotion of government policies; (3) GDP (constant prices in 1990): the average error is 2.67%, with the actual value larger than the simulated. It indicates that the Chinese economy grew faster than expected during this period. Especially in 2007, 2008 and 2015, the real GDP is more than 5.0% higher than the simulated.

From the perspective of the three industries, the actual value of output value of primary industry is less than the simulated with an average error of 7.29%, especially over 5.0% between 2007 and 2009 and more than 10.0% between 2000 and 2006. It indicates that the agricultural sector in this period showed a significant negative growth. At the same time, the actual value of the output value of secondary industry is greater than the simulated, with an average error of 5.53%, greater than 7.0% during 2006-2011, and more than 10.0% during 2007-2008, indicating that the industrialization process has accelerated significantly during this period.

This trend is also seen in terms of energy production. In general, the actual output is larger than the simulated amount, with an average error of 7.6%, especially between 2004-2008 and 2010-2011 and 2015 being greater than 10.0%. Since 1998, energy production has achieved the goal of "advance in quantity and ensure supply", compared with production in the industrial sector. Because China’s coal resources are abundant and the number of private coal companies is large, the actual coal output is much larger than the simulated. During 2000-2002 and 2013 and 2015, the actual output exceeded the simulated by 6.9- 9.8%, especially more than 10.0% from 2004 to 2012.

Because state-owned enterprises control oil resources and mining in China, little difference exist between actual and simulated outputs, with an average error of only 1.56%. Natural gas, a new type of energy in China, is greatly affected by national policies. The actual output is larger than the simulated, with an average error of 7.33%. In the years of rapid economic growth such as 2006 and 2011, such error was 11.0–13.6%. In 2007–2010 it exceeded 15.0%, and in 2008 it even reached 20.17%. For Non-fossil energy production, the actual output is smaller than the simulated, with an average error of 3.6%, especially 8.7% less in 2011, indicating that the development of non-fossil energy is not as good as expected. In terms of energy consumption, the actual output is mainly larger than the simulated, with an average error of 6.12%. Except 11.0–15.0% during 1998–1999 and 5.0% between 2004–2006 and 2008, the others were less than 5%.

From the perspective of primary, secondary, tertiary industries energy consumption, the overall actual consumption is larger than the simulated, with an average error of 7.04%. Especially during the rapid economic growth of 1998–1999 and 2004–2006, the actual consumption was 10.0–15.97% more than the simulated. Even in the financial crisis of 2007–08, the actual consumption was 8.4% more than the simulated. However, the actual consumption was 18.8% less than the simulated since 2015. China’s production and economy have entered a low-growth stage, reflected by Northeast China and Shandong Province’s economic recession and shrinking energy demand. Except that the actual consumption of 2002 is less than 10.39% of the simulated, the actual energy consumption of residents in most years is larger than the simulated, with an average error of 3.81%. In terms of energy consumption (tce), the errors in most years are within 4%, with the average error being 5.19%. The continued growth of energy in the areas of residential consumption and transportation is actually consistent with the fact of accelerated urbanization promoted by the government since 2000.

### Results 2

#### The level of urbanization

**Figure 4:**
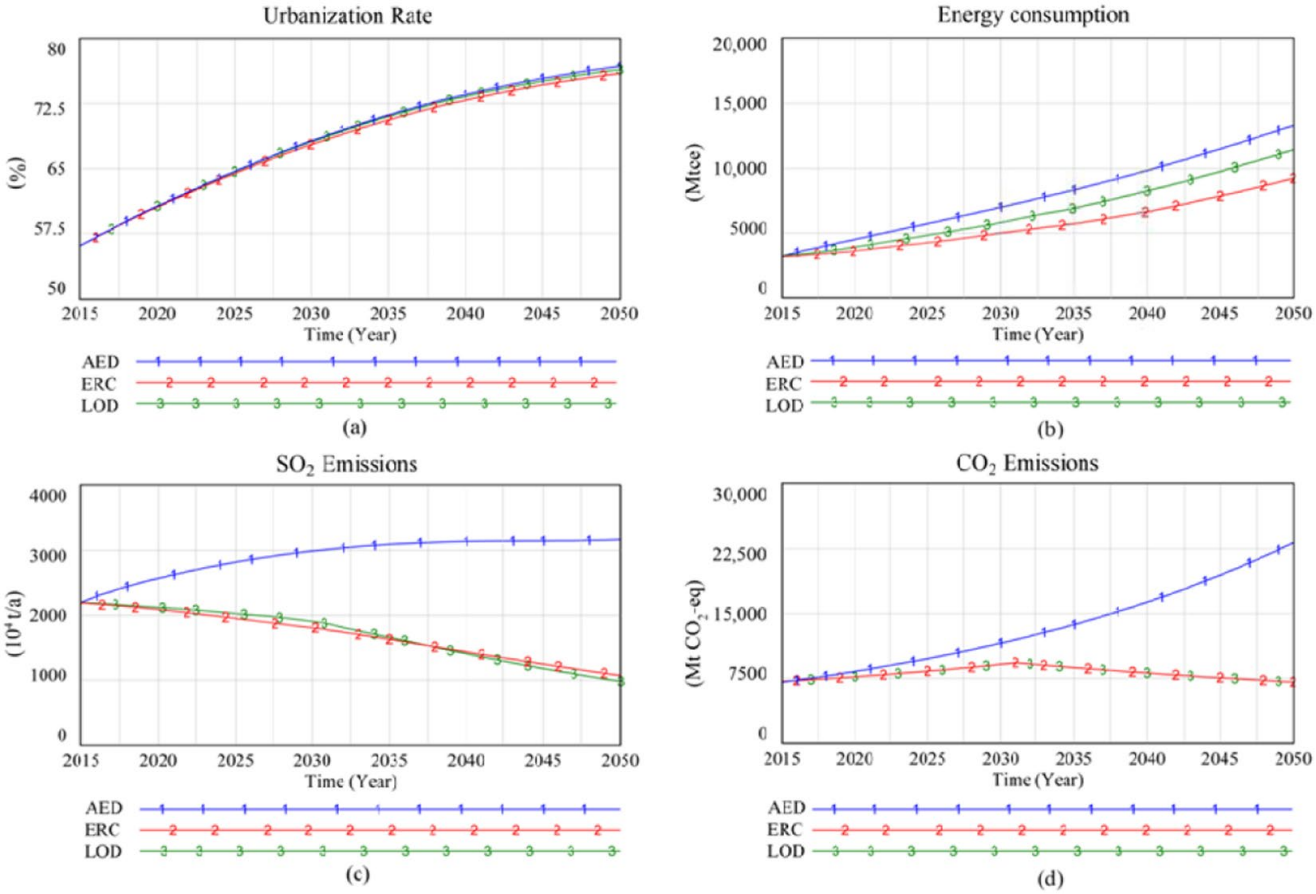
Comparison of simulation results for three development scenarios.

Three alternative policy scenarios are implemented into the system simulation, and the corresponding urbanization level is shown in Fig. 4(a) and Table 5. In the case of the AED scenario, the total GDP volume will increase from 19.7 trillion Yuan in 2015 to 221.1 trillion Yuan in 2050, with an annual growth rate of 7.1%. China’s urbanization level will reach 70.0% in 2035 and 76.79% in 2050. However, economic growth rate will slow down to reduce energy demand substantially in the ERC scenario. By 2020, GDP growth rate will drop by 13.79% compared to the AED scenario. Over time, this gap will continue to expand. By 2050, GDP growth rate under the ERC scenario is only 57.72% of the AED scenario. The growth rate of China’s urbanization will drop with the slowdown of economic growth even though in this case scenario. The urbanization level will reach 69.59% in 2035, and 75.96% in 2050. Under the LOD scenario, non-fossil energy growth goes a long way towards speeding up the total energy supply and boosting urbanization and economic development. Therefore, compared with the ERC scenario, the urbanization level in this scenario is projected to be 76.41% in 2050.

**Table 5:** Simulation values of three scenarios of Chinese Urbanization-energy SD model.

		AED scenario				ERC scenario				LOD scenario			
		2015	2020	2035	2050	2015	2020	2035	2050	2015	2020	2035	2050
Total GDP volume (trillion Yuan)		19.72	28.78	89.37	221.10	19.72	24.81	42.64	127.63	19.72	26.67	71.63	171.97
Urbanization level (%)		56.09	60.67	70.00	76.79	56.09	60.56	69.59	75.96	56.09	60.66	71.00	76.41
Total amount of energy consumption (Mtce)		4126.16	4521.54	8353.86	13313.46	4126.16	4238.78	5750.57	9238.31	4126.16	4407.88	6929.79	11416.15
Industrial energy consumption	(Mtce)	3219.48	3370.45	5841.15	8225.31	3219.48	3209.56	3658.51	5784.85	3219.48	3321.66	4742.39	7094.85
	(%)	78.03	74.54	69.92	61.78	78.03	75.72	63.62	62.62	78.03	75.36	68.43	62.15
Residential energy consumption	(Mtce)	508.46	699.89	1893.74	4425.74	508.46	611.33	1301.38	2816.01	508.46	658.74	1678.91	3682.58
	(%)	12.32	15.47	22.67	33.24	12.32	14.42	22.63	30.48	12.32	14.94	24.23	32.26
Transportation energy consumption	(Mtce)	398.22	451.20	618.98	662.41	398.22	417.89	579.62	637.45	398.22	427.48	605.62	638.72
	(%)	9.65	9.98	7.41	4.98	9.65	9.86	10.08	6.90	9.65	9.70	8.74	5.59
Coal	(Mtce)	2610.02	3084.94	3646.27	8411.29	2610.02	2673.21	2707.86	2332.90	2610.02	2673.21	2707.86	2332.90
	(%)	72.10	71.03	58.89	60.78	72.10	67.13	51.83	35.97	72.10	65.95	45.34	23.47
Oil	(Mtce)	307.70	331.48	414.43	518.13	307.70	388.10	558.42	321.87	307.70	388.10	558.41	321.87
	(%)	8.50	7.63	6.69	3.74	8.50	9.75	10.69	4.96	8.50	9.57	9.35	3.24
Natural gas	(Mtce)	177.38	239.62	590.73	1456.34	177.38	233.71	417.59	378.37	177.38	233.71	417.59	378.37
	(%)	4.90	5.52	9.54	10.52	4.90	5.87	7.99	5.83	4.90	5.77	6.99	3.81
Non-fossil energy	(Mtce)	524.90	687.00	1540.3	3453.30	524.90	687.00	1540.3	3453.3	524.90	758.48	2288.5	6904.87
	(%)	14.50	15.82	24.88	24.95	14.50	17.25	29.48	53.24	14.50	18.71	38.32	69.48
CO_2_ emissions (billion tons)		7.15	8.31	13.73	23.22	7.15	7.69	8.62	7.07	7.15	7.69	8.76	7.07
SO_2_ emissions (Mt)		21.97	25.68	30.92	31.67	21.97	17.18	16.29	10.70	21.97	19.77	16.57	9.79

#### Energy demand and supply

Simulation results of total energy consumption and energy demand of different sectors are illustrated in Fig. 4(b) and Table 5 respectively. The total amount of energy consumption will increase from 4126.16 Mtce in 2015 to 13313.46 Mtce in 2050, with an increase of 2.23 times under the AED scenario. Energy consumption of the primary, secondary, and tertiary industries greatly contributes to the final energy consumption. Due to the transformation of the heavy chemical industry and technological advancements, energy consumption per unit GDP will drop gradually and the growth rate of the industry energy consumption tend to slow down. The proportion of the industrial energy consumption will decrease from 78.03% in 2015 to 61.78% in 2050, while the proportion of residential energy consumption will rise from 12.32% in 2015 to 33.24% (4425.74 Mtce) in 2050. The total amount of transportation energy consumption will reach 662.41 Mtce, 1.66 times that of 2015.

In the case of the ERC scenario constrained by the fossil energy supply, the total amount of energy consumption will be 9238.31 Mtce, 30.45% less than that of the AED scenario in 2050. It is almost 63% in 2050 where the proportion of energy consumption of the primary, secondary, tertiary industries account for total energy consumption. While compared with AED scenario, a decrease of 3.35% for the proportion of residential energy consumption accounting for the total amount of primary energy consumption, arrives at 30.05% in 2050.

Under the LOD scenario, the total energy consumption will be 11416.15 Mtce in 2050. Among them, the residential energy consumption will reach 3682.58 Mtce, whose proportion accounting for the total amount of primary energy consumption increased by 1.78% in 2050 compared to ERC scenario. The proportion of transportation energy consumption accounting for the total energy consumption will decline 1.31%, due to the policy of the ban on fuel automobiles.

The structural changes of energy supply from 2015 to 2050 are listed in Table 5. The energy usage structure of China varies across scenarios. In the case of AED scenario, the supply of coal in 2050 will be 3.22 times that of 2015, showing a robust growth trend. It still leads the portion of energy supply, reaching 61.78% in 2050. The total oil supply increases but with a declining share in the energy structure. The supply of oil increases but with a flat growth rate. The supply of natural gas soars rapidly, accounting for 10.5% of energy supply in 2050. The non-fossil energy will increase from 14.47% in 2015 to 25.1% in 2050.

Regarding the energy supply structure of ERC scenario, the proportion of all kinds of fossil energy accounting for total energy supply will drop tremendously. Among them, the proportion of coal supply to the total energy supply in 2050 is 35.97%, a decrease of 36.13% than that in 2015. The proportion of oil and gas in the energy supply are 4.96% and 5.83% in 2050, respectively. The supply of fossil energy will reach its peak in 2030. Then, with the gradual decrease of domestic supply, the dependence on external energy will increase, reaching 23.7% in 2050.

As for the LOD scenario, the supply volume of coal, oil, and natural gas will be almost the same with that under the ERC scenario. However, due to the significant growth of non-fossil energy supply, the proportion of fossil energy in the energy structure will further decrease. The major difference between ERC and LOD scenarios is that they make different assumptions about China’s future energy structure. Although the amount of fossil energy in the two scenarios is the same, the total energy supply in the LOD scenario is 37.36% higher than the ERC scenario, due to the substantial increase in non-fossil energy production in the LOD scenario. It can better meet China’s socioeconomic development needs. Correspondingly, the external energy dependence has also decreased from 29.78% in the ERC scenario to 14.8%, ensuring national energy security.

#### Environmental effects of energy

The CO_2_ and SO_2_ emission values of different scenarios are listed in Fig. 4(c,d), and Table 5, respectively. Due to the continuous and rapid growth of China’s energy consumption and the dominating role of fossil energy in the energy structure, the CO_2_ emissions from energy consumption will increase from 7.05 billion tons in 2015 to 23.26 billion tons in 2050. It will exacerbate China’s CO_2_ emission reduction pressure in the AED scenario. The SO_2_ emissions from coal combustion will be 31.67 Mt in 2050, another major threat to the environment. However, in the ERC and LOD scenarios, the total amount of fossil energy is controlled. By 2050, the CO_2_ emissions caused by energy consumption will be reduced by 69.55% compared to the AED scenario, which is only 7.07 billion tons, equivalent to the 2015 level. CO_2_ emissions per 10,000 yuan of GDP in 2020 will be only 28.87 tons. SO_2_ emissions in the ERC scenario are reduced to 10.70 Mt, only 33.78% of that in the AED scenario. Since the SO_2_ removal rate of per unit coal is increased by 2.3% more than that in the case of ERC, the SO_2_ emissions of the LOD scenario will drop to 9.79 Mt^85^.

In summary, the rapid economic growth and sufficient energy supply will accelerate the development of China’s urbanization in the case of AED scenario. However, this mode not only ignores the fact that the growth of fossil energy supply capacity is limited, but also imposes huge environmental pressures. While the ERC scenario will greatly reduce CO_2_ emissions, it also hurdles fast economic growth and urbanization. Moreover, China will face the risk of over dependence on external energy, with the increase of the gap between energy supply and demand. It could be safely concluded that an ideal mode to achieve a healthy and rapid development of urbanization is clearly illustrated in the case of low carbon oriented development, which is to prioritize the renewable energy development, optimize energy structure, as well as improve household lifestyle. It will achieve CO_2_ emission reductions, environmental protection, and the sustainable socio-economic development.

## Discussion and Conclusion

The trends of China’s economy, urbanization, and energy structure have been widely studied recently (Table 3 in Appendix C). Annual growth rate of GDP is estimated to be 5.5–7.0% in 2010–2035, 4.0–6.0% in 2035–2050, and below 3% after 2050. The urbanization level will reach 70–72% in 2035, and above 75% in 2050. The total amount of energy consumption (Mtce) will reach 5000–6000 Mtce in 2035, and 6000–6500 Mtce in 2050. Coal consumption will drop from 63.0% to less than 45%, oil from 20% to about 15%, natural gas from 5.5% to about 12%, and non-fossil energy from about 10% to about 30% by 2050. CO_2_ emissions will also drop from about 8.0 billion tons to about 5.0 billion tons by 2050. According to the simulation of energy-urbanization SD model, the GDP growth rate is set (the primary industry growth rate of 3–4%; the secondary industry growth rate of 5–7%; the tertiary industry growth rate of 6–8%) to achieve the urbanization rate of 75.0–80.0%. The total amount of energy consumption (Mtce) will reach 5750.57–8353.86 Mtce in 2035, and 9238.31–13313.46 Mtce in 2050. Non-fossil energy will be about 65% in 2050, and CO_2_ emissions will be about 7.07 billion tons in 2050.

Energy is not only the necessary driver for China’s urbanization but also the constraint factor in urbanization for fulfilling the mission of global CO_2_ emission reductions^86,87^. In order to estimate the urbanization development, energy demand and environmental status in China over 2015–2050, an integrated SD model composed of four sub-models has been developed^88^. The validity of the model has been confirmed by the simulation and sensitivity analysis using the data of 1998–2015, providing strong evidence that the effective energy planning and management policies are needed for the low-carbon oriented urbanization.

Hence, “a research priority entails undertaking new integrated studies that aim to close the loops: fully connect the socioeconomic and environmental systems and understand their dynamic interplay and feedback”^16^. This paper explores the implications of three urbanization models for energy consumption and emissions: accelerated economic development, emission reduction constraint, and low-carbon oriented scenarios. Our results show that in the case of accelerated economic development, the urbanization level in China might reach 76.79%, in spite of facing exacerbating environmental damage and increasing emission reduction pressure. CO_2_ emissions in China will arrive at 7.07 billion tons in the case of emission reduction constraints, but will result in negative effects on the potential speed of China’s economic development and urbanization, a decline of GDP by 42.27% and urbanization by 0.83% in 2050. The low carbon-oriented development is ideal for sustainability by combining rapid urbanization (the urbanization level in China will reach 76.41% in 2050), a low carbon emission target, and eco-friendly environment. The industrial energy consumption serves as a significant driving force for China’s urbanization in all three scenarios. The urbanization leads to a rapid growth of residential energy consumption, accounting for 30.48–33.31% of total energy consumption in 2050 as the second largest energy consumption sector after industrial energy consumption. China’s energy demand of non-fossil energy production capacity will grow from 14.5% of energy demand in 2016 to 53.24–69.48% in 2050.

To achieve a paradigm shift towards low-carbon transition associated with rapid urbanization, the following key steps should be taken: (1) the reduction of energy intensity would be facilitated by promoting scientific and technological innovation. It is urgent to improve the technological innovation in all industries that will advance the energy intensity reduction and cut down CO_2_ emissions. (2) Low carbon household units are crucial in achieving low-carbon transition of urbanization. Although the gap between the energy consumption per capita in China and developed countries is still large, the top 10% individuals have reached the average per capita energy consumption in developed countries. In the next 10 to 20 years, the challenges of energy, environment, and adaptation to climate change will challenge Urban China’s social and economic development. In particular, China's current urbanization model is associated with excessive energy consumption and environmental damage. With the scientific and technological advances, both energy intensity of the three industries and the proportion of industrial energy consumption in total consumption will also decrease. With the growth of urbanization rate, the size of the urban population has gradually increased, and the per capita energy consumption in urban areas is much higher than that in rural areas. Faced with the increasingly severe resource and environmental issues, it is critical to implement a low-carbon urbanization transition. On the one hand, the development of a low-carbon economy can avoid carbon lock-in and path dependence as well as the emergence of high energy-consuming industrial production and urban infrastructure. On the other hand, controlling urban energy consumption is important for China to deal with the increasingly serious energy crisis and climate change. It is needed to guide household energy consumptions, and actively promote the use of solar power, wind power, biogas, and other clean renewable energy. (3) It is vital to adopt a series of technical and institutional measures to advance new energy development. Priorities should be given to establish non-fuel energy supply capacity. It is also important to set up the integrated energy supply system. In addition, we need to recognize the geographical variance^12^.

The scenarios in this paper are three most possible paths in China. We acknowledge the existence of other scenarios such as: (a) no consideration of a scenario with robust economic development and a concurrent shift to low-carbon resources and (b) a low-carbon development scenario that does not include very large growth in production of oil and gas (and to a lesser degree coal). We will investigate these topics in the follow-up studies^76,77,89–119^.
